# Molecular identification of *Mycobacterium bovis* from cattle and human host in Mali: expanded genetic diversity

**DOI:** 10.1186/s12917-016-0768-7

**Published:** 2016-07-20

**Authors:** Mamadou Diallo, Bassirou Diarra, Moumine Sanogo, Antieme C. G. Togo, Anou M. Somboro, Mariam H. Diallo, Bréhima Traoré, Mamoudou Maiga, Younoussa Koné, Karim Tounkara, Yeya dit Sadio Sarro, Bocar Baya, Drissa Goita, Hamadoun Kassambara, Bindongo P. P. Dembélé, Sophia Siddiqui, Robert L. Murphy, Sounkalo Dao, Souleymane Diallo, Anatole Tounkara, Mamadou Niang

**Affiliations:** Laboratoire Central Vétérinaire, Bamako, Mali; SEREFO, University of Sciences, Techniques and Technologies of Bamako (USTTB), Point-G, Bamako, Mali; Division of Clinical Research, NIH, Bethesda, USA; Northwestern University, Chicago, IL USA

**Keywords:** Bovine tuberculosis, Frequency, Spoligotyping, Mali

## Abstract

**Background:**

Bovine tuberculosis (BTB) is a contagious, debilitating human and animal disease caused by *Mycobacterium bovis*, a member of the *Mycobacterium tuberculosis* complex. The study objective were to estimate the frequency of BTB, examine genetic diversity of the *M. bovis* population in cattle from five regions in Mali and to determine whether *M. bovis* is involved in active tuberculosis (TB) in humans. Samples from suspected lesions on cattle at the slaughterhouses were collected. Mycobacterial smear, culture confirmation, and spoligotyping were used for diagnosis and species identification*. Mycobacterium* DNA from TB patients was spoligotyped to identify *M. bovis*.

**Results:**

In total, 675 cattle have been examined for lesions in the five regions of Mali. Out of 675 cattle, 79 specimens presented lesions and then examined for the presence of *M. bovis*. Thus, 19 (24.1 %) were identified as *M. bovis*; eight (10.1 %) were non-tuberculous *Mycobacterium* (NTM). Nineteen spoligotype patterns were identified among 79 samples with five novel patterns. One case of *M. bovis* (spoligotype pattern SB0300) was identified among 67 TB patients.

**Conclusion:**

This study estimates a relatively true proportion of BTB in the regions of Mali and reveals new spoligotype patterns.

## Background

Bovine tuberculosis (BTB) is a contagious, debilitating human and animal disease. It is caused by *Mycobacterium bovis* a member of the *M. tuberculosis* complex (MTBc), which also includes *M. tuberculosis, M. africanum, M. microti, M. canettii, M. caprae*, *M. pinnipedii* and the new discovered strain *M.mungi* [1]*.* Cattle are considered as natural reservoir for *M. bovis*.

In Mali, West Africa, the total number of cattle is estimated to be more than 9,438,000, and the livestock sector occupies an important place in the development of the economy [2]*.* In terms of the national economy, the livestock sector contribution to gross domestic product (GDP) is estimated to be 10 % and its contribution to export earnings was 40 billion CFA ($83 million United States of America Dollar: USD) in 1995, which represents 17.5 % of total exports, occupying third place after cotton and gold [3]*.* The economic contributions from cattle have steadily declined in recent years, and were only 28 billion CFA ($58 million USD) in 1999 representing 7 % of total exports [4]*.*

The monitoring of BTB is based partially on screening by tuberculin skin test of live animals and also on the testing of suspected lesions found at slaughterhouses. Bovine tuberculosis is a zoonotic disease caused by *M. bovis*, which is transmitted, from animals to humans by ingestion of raw milk, contaminated meat or aerosol [5]. *Mycobacterium bovis* infection in humans often presents as extra pulmonary tuberculosis, especially in children [5]*.* The possible role of *M. bovis* in human tuberculosis infection is not well known in Mali because tuberculosis in humans due to *M. bovis* is clinically indistinguishable from that of *M. tuberculosis.*

In many resource-limited settings, the diagnosis of tuberculosis is based only on smear microscopy which cannot differentiate *Mycobacterium* species or the different strains of MTBc. Bovine tuberculosis cannot be specifically diagnosed without confirmation using additional microbiological methods. New molecular methods such Mycobacterial Interspersed Repetitive Units (MIRU), Variable Number of Tandem Repeats (VNTR) and restriction fragment length polymorphism (IS*6110* RFLP) techniques [6] can determine the importance and factors influencing the transmission of BTB among cattle. Among these methods, spoligotyping technique is most often used for molecular typing of *Mycobacterium* strains [7]. This technique has the advantage to confirm the diagnosis of tuberculosis and also to classify subspecies of *Mycobacterium tuberculosis* complex (MTBc) [7]*.* Because of this method, *M. bovis* was isolated in countries where mycobacterial isolates from humans were fully typed [7].

In Mali, there has been an increase in the number and variety of cattle, and as well as in the number of tuberculin skin test-positive cases. Several studies on *M. bovis* population focused only in the capital city of Bamako have been performed excluding other regions of Mali [8, 9]. The goal of this study was first to estimate the apparent prevalence of BTB, second to examine the genetic diversity of strains of *M. bovis* obtained in five distinct regions of Mali from infected cattle, using spoligotyping technique*.* Lastly, we examined whether *M. bovis* is involved in active TB in the human host.

## Methods

### Slaughterhouses and specimens collection

The regions of Ségou, Mopti, Sikasso, Kayes and Bamako were selected for this study. These five regions are home to more than 80 % of the livestock of Mali [4]*.* In each region there is a slaughterhouse maintained by a trained veterinarian.

We conducted this study from January 2008 to December 2010. During that period, 675 cattle were examined for the presence of lesions in the regions of Kayes (75), Sikasso (160), Ségou (80), Mopti (140) and Bamako (220). At the slaughterhouses, specimen collection was based on suspicion of bovine tuberculosis lesions after slaughtering by meat inspection [10]. Suspected lesions were lymph nodes and or necrotic nodules. They were size different and were containing yellow, green or tan pus. Tissue specimens were collected from affected organs such as lymph nodes, lungs, chest, and liver as described by Cosins [9]*.* Upon collection, tissue specimens were transported on ice to the Central Veterinary Laboratory of Bamako (Laboratoire Central Vétérinaire de Bamako) and kept at −80 °C before testing. Each specimen was uniquely identified by collection date, sample number and region of origin.

### Specimens processing, smear microscopy and culture

All the laboratory work was performed in the BSL-3 facility within the Human Immunodeficiency Virus and Tuberculosis (HIV-TB) Research and Training Center (SEREFO Laboratories) at the University of Sciences, Techniques and Technologies of Bamako (USTTB).

Tissue specimens were washed with sterile saline solution (Remel Inc., Lenexa, KS 66215, USA). Specimens were then cut into small pieces using the Tissue Grinder (Precision Disposable Tissue Grinder, Covidien, Mansfield, MD, USA). Thereafter, specimens were digested and decontaminated using the standard N-Acetyl-L-Cysteine/4 % NaOH solution (Alpha Tec System. Inc., Vancouver, USA), concentrated by high speed centrifugation (4500 rpm or 3000 g, Eppendorf centrifuge) and inoculated on both liquid (Mycobacterium Growth Incubator Tube, BBL™ MGIT™ Becton Dickinson, Sparks MD, USA), and solid (Middlebrook 7H11 Agar and Selective 7H11 Agar) media. Simultaneously, an aliquot of concentrated specimen was prepared for Auramine-Rhodamine staining (BBL™ Becton Dickinson, Sparks MD, USA) for microscopic examination (Olympus, Olympus Corporation, Tokyo, Japan).

### Identification of mycobacterial isolates

Positive cultures were confirmed by smear using Kinyoun (Remel Inc. Lenexa, KS 66215, US) stain acid fast method. The mycobacterial infection of specimens was based on a combination of the initial fluorescent acid-fast microscopy result, colonial morphology of growth in culture, and confirmation by Kinyoun staining. Suspected tuberculosis cases were confirmed by spoligotyping technique.

In order to test whether there is transmission of *M. bovis* in human hosts, we examined the spoligotyping results for 67 new human TB cases screened at the University Teaching Hospital of Point G and the six-TB care units located in the health referral centers of Bamako between 2006 and 2010 from SEREFO database.

### Molecular strain typing for genetic diversity study within M. bovis

All Kinyoun-positive colonial growth suggestive of *M. bovis* underwent molecular strain typing. Spoligotyping technique was performed using a commercially available kit (Isogen Life Science, Netherlands) by following the manufacturer’s instructions to amplify the whole direct repeat region (DR) within the *M. tuberculosis* genome. The amplified biotin labeled PCR product was then hybridized to 43 ordered synthetic DNA oligonucleotide spacers bound to a nylon membrane. After hybridization, the membrane was developed by incubating with a streptavidin peroxidase conjugate, which binds to the biotin label on the PCR product. Next, the membrane was washed and incubated with Enhanced Chemiluminescent (ECL) Detection Reagents. This reaction results in the emission of light, and is detected by exposing a light sensitive film to the membrane. Comparison and naming of the patterns were done through global *M. bovis* database (http://www.Mbovis.org).

### Statistical analysis

The proportion of each strain type with 95 % confidence interval (CI) and their geographical region were compared using the *χ*^2^ test or Fisher’s exact test. The study included all the suspected cases which were seen at each slaughterhouse during our visit. A descriptive analysis was performed by evaluating the number of isolates determined to be *M. bovis cases* by spoligotyping technique. Thereafter similar spoligotypes were grouped in a same cluster. The relationship of clusters to geographical origin of animal was also evaluated. At the same these patters were compared to the *M.bovis* case which was isolated from human during one of our previous study on human tuberculosis.

## Results

**Fig. 1:**
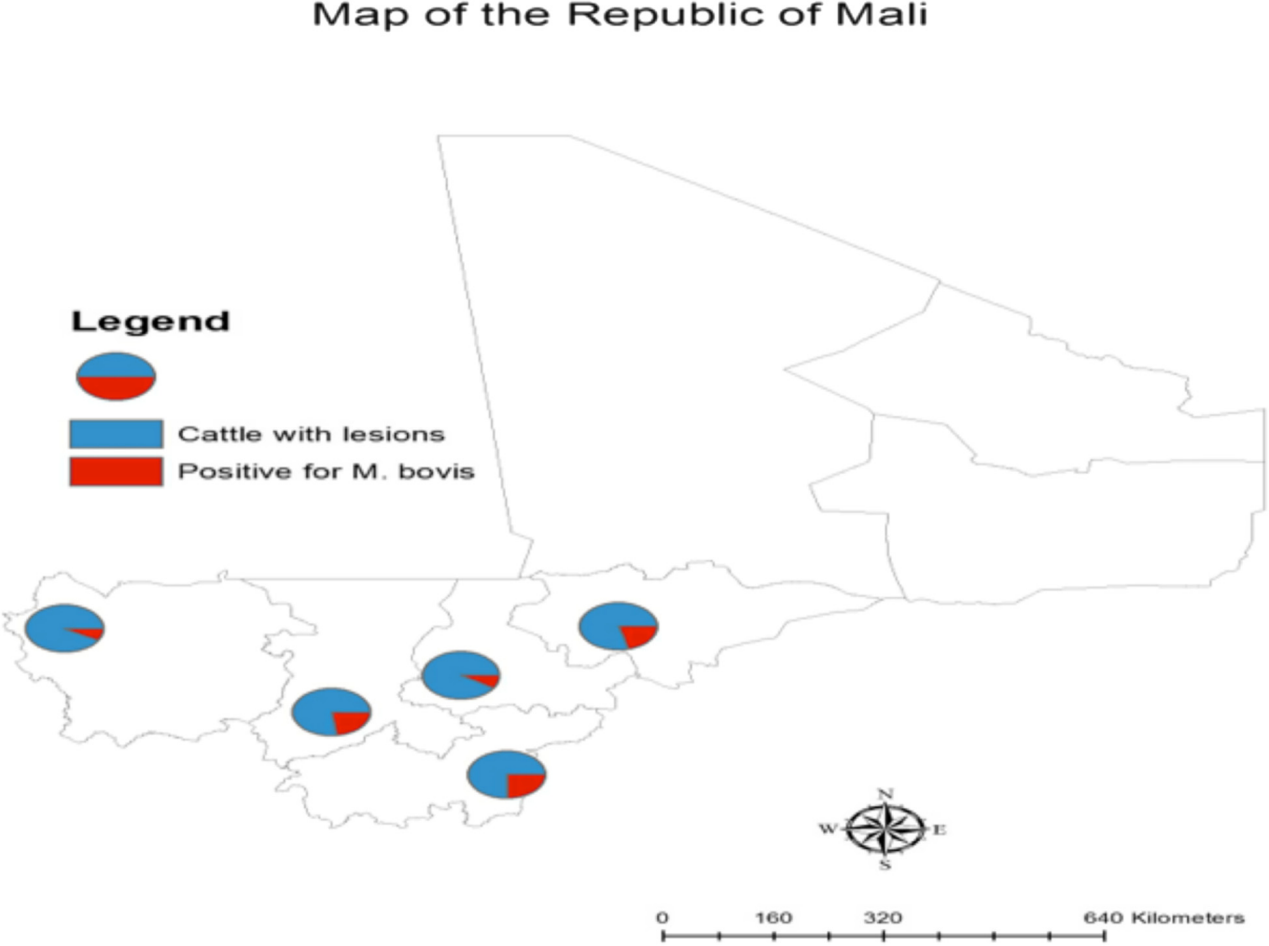
The map of the Republic of Mali. The blue represents the number of cattle with confirmed *M.bovis* in each studied region and the red indicates the number of cattle with *M. bovis* in each studied region. The higher numbers of cattle with *M. bovis* infection have been found in Bamako, Sikasso and Mopti. (*Thanks to Dansine Diarra, Bamako, Mali for making this map of Mali*)

During the study period, in total 675 cattle were examined in the 4 regions and the district of Bamako where the study was conducted (Fig. 1).

Among the 675 cattle screened between January 2008 and December 2010, 79 have presented lesions leading to a rate of 11.7 % [95 % CI 9.3–14.4] (Table 1). The regions of Kayes and Mopti have presented the highest rate of lesions respectively 14.7 % and 13.6 %. In contrast, Sikasso has the lower rate with 7.5 % (Table 2). The general frequency of *M. bovis* infection was 2.81 % with the highest prevalence in Bamako (8/675) with 1.18 % and the lowest was observed in Kayes and Sikasso (1/675) with 0.15 % (Table 1). Tests performed on the 79 lesions obtained, the number of infected lesions with *M. bovis* was observed in Sikasso, Bamako and Mopti with respectively 33.3, 29.6 and 26.3 % (Table 2). The mean age of the cattle was 4 years old, and the majority were female.

**Table 1:** Tissue sample number, region of provenance and prevalence using liquid media technique

Sample provenance	Number of examined cattle	Number of cattle with lesions	Percentage of cattle with lesion	Number of positive *M. bovis*	Percentage of positive (microscopy)	Percentage of positive (liquid media culture)
Kayes	75	11	14.7 %	1	1/11 (9.1 %)	1/11 (9.1 %)
Sikasso	160	12	7.5 %	4	4/12 (33.3 %)	4/12 (33.3 %)
Ségou	80	10	12.5 %	1	0/10 (0 %)	1/10 (10 %)
Mopti	140	19	13.6 %	5	4/19 (21.55)	5/19 (26.3 %)
Bamako	220	27	12.3 %	8	7/27 (25.9 %)	8/27 (29.6 %)
Total	675	79	11.70	19	16/79 (20.25 %)	19/79 (24.05 %)

Among the 675 cattle screened, 79 have presented lesions leading to a rate of 11.70 %. The regions of Kayes and Mopti have presented the highest rate of lesions respectively 14.7 % and 13.6 %. In contrast, Sikasso has the lower rate with 7.5 %. The general prevalence of the infection was 2.81 % with the highest prevalence in Bamako (8/675) with 1.18 % and the lowest was observed in Kayes and Sikasso (1/675) with 0.15 %. Tests performed on the 79 lesions obtained, the number of infected lesions with *M. bovis* observed was higher in Sikasso, Bamako and Mopti with respectively 33.3, 29.6 and 26.3 %. This table shows that 34.2 % of cattle were investigated in Bamako followed by Mopti with 24.1 % and Ségou has the lowest number of cattle 12.6 %. This was no statistically significant difference between the number of specimens collected in each site (*p* = 0.47)

**Table 2:** Proportion of infection per region

Sample provenance	Number of examined cattle (%)	Number of Positive (*M. bovis*) by microscopy and (%)	Number of Positive (*M. bovis*) by liquid media culture (%)
Kayes	75 (11.1)	1/75 (1.33 %)	1/75 (1.33 %)
Sikasso	160 (23.7)	4/160 (2.5 %)	4/160 (2.5 %)
Segou	80 (11.9)	0/80 (0 %)	1/80 (1.25 %)
Mopti	140 (20.7)	4/140 (2.86 %)	5/140 (3.57 %)
Bamako	220 (32.6)	7/220 (3.18 %)	8/220 (3.63 %)
Total	675	16/675 (2.37 %)	19/675 (2.81 %)

The estimated prevalence (stated) as proportion of infection of *M. bovis* was 2.37 % (16/675) by microscopy and 2.81 % (19/675) using the liquid media culture. The gold standard test is liquid media culture. There is disparity among regions, the highest prevalence (culture) was in Mopti (3.57 %) and Bamako (3,63 %) and the lowest was in Segou (1.25 %)

Using the culture technique, 19 specimens out of 79 were confirmed as BTB (24.1 %) (Table 3). Eight (10.1 %) cultures positive for acid-fast bacilli were found to be non-tuberculous mycobacteria (NTM) strains, while 23 samples (29.1 %) were contaminated with other bacteria or yeast (Table 1). The sequencing of the 16sRNA to identify the species of those bacteria was not performed.

**Table 3:** Culture results of tissue specimens from Malian cattle. Specimens were digested, decontaminated, centrifuged at high speed and inoculated onto two types of media

Tissue specimens culture results	Number (*N*)	Percentage (%)
Positive (*M. bovis*) Positive (NTM) Negative Contaminated with other bacteria	19 8 29 23	24.1 10.1 36.7 29.1
Total	79	100

Using the culture system, 27 of 79 were positive; one can notice that 19 were *M. bovis* (24.1 %) while eight were Non Tuberculous Mycobacteria (10.1 %). The contamination with other species of bacteria has been estimated

Spoligotype patterns were assessed during the study. The absence of spacers 3, 9, 16 and 39–43 was used to distinguish *M. bovis* [7]. Our data showed that all 19 specimens investigated were identified as BTB and lacked these spacers (Table 4)*.* Comparison of the patterns with the global *M. bovis* database revealed five spoligotypes that had not previously been reported (SB2262, SB2263, SB2264, SBXXXX mopti and SBXXXX bamako) in Mali and elsewhere (Table 4). Those five were subsequently registered in the *M. bovis* database in http://www.mbovis.org.

**Table 4:** Spoligotype patterns revealed during the study in Mali and their frequency

SB number	Frequency	Percent	Pattern	Region
SB2262^a^	2	10.52	1101101101111100111101111111101111111100000	Bamako
SB2263^a^	3	15.78	1101101101111110111101111111101101111100000	Bamako
SB2264^a^	1	5.26	1101101101111110111111111111101011111100000	Bamako
SB0300	6	31.57	1101101101111110111111111111101111111100000	Kayes, Bamako, Ségou, Mopti
SB1275	1	5.26	1101111101111110111101111111101111111100000	Sikasso
SBXXXX	1	5.26	1100011101111110111110011111101111111100000	Mopti
SBXXXX	2	10.52	1101101101111110111111111111101011111100000	Bamako
SB0944	3	15.78	1101111101111110111111111111101111111100000	Sikasso
Total	19	100		

^a^Three new patterns have been identified, SB2262, SB2263 and SB2264 and are found only in Bamako. The SB1275 and SB0944 are found in Sikasso. In contrast, the SB0300 is found in all sites except Sikasso. The frequency of the SB2262 pattern is more frequent in Bamako with 36.84 % followed by the SB0300 pattern with 31.57 % in the four of the five study sites

**Fig. 2:**
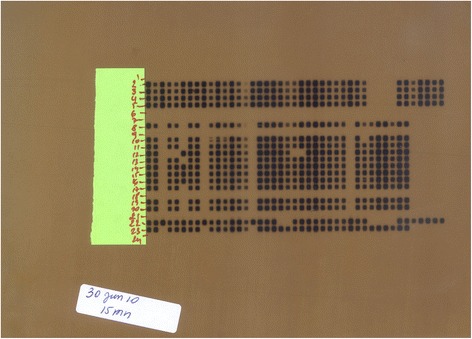
Spoligotyping test run on 30 Jun 2010 (*30 Jun 10*). 1 = Buffer. 2 = MAL02020101. 3 = MAL02020201. 4 = MAL02020501. 5 = MAL02020801. 6 = LCV-1. 7 = LCV-7. 8 = LCV-9. 9 = LCV-10. 10 = AFS-2. 11 = MMP-1. 12 = MMP-2. 13 = MMP-3. 14 = SKSS-1. 15 = SKSS-3. 16 = SKSS-4. 17 = SKKL-1. 18 = SKKL-2. 19 = SgSg-2. 20 = Mate-1. 21 = Negative control. 22 = Positive control-1 (H37Rv). 23 = Positive control-2 (*M.bovis*). 24 = Buffer. Numbers represent the patients Laboratory unique ID or Tissues Identification numbers

**Fig. 3:**
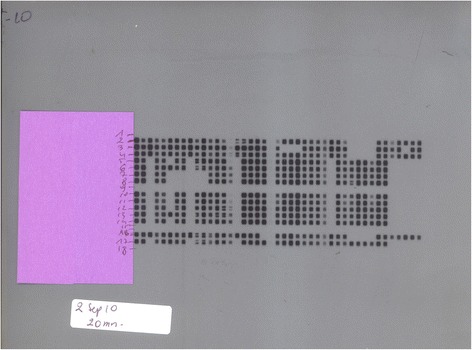
Spoligotyping test run on 2 September 2010 *(2 Sep 10*). 1 = Buffer. 2 = MAL02020601. 3 = MAL02020901. 4 = MAL020201101. 5 = Bko-2. 6 = SKSS-2. 7 = AFS-1. 8 = AFS-3. 9 = LCV-7. 10 = MMP-2. 11 = Bko-4. 12 = Bko-1. 13 = SKSS-2. 14 = Bko-3. 15 = Negative control. 16 = Positive control-1 (H37Rv). 17 = Positive control-2 (*M.bovis*). 18 = Buffer. Numbers represent the patients Laboratory unique ID or Tissues Identification numbers

There was a disparity in the distribution of spoligotypes among the five sites. Among the newly identified spoligotype patterns, the SB2262 was the most frequent with 36.84 % and found only in Bamako. The spoligotype pattern SB1275 was present only in Sikasso (in the south of the country at the Côte D’Ivoire border) as well as the SB0944, which represented 15.78 % of all the patterns (Table 4). The SB0300 was widely spread throughout the country (Bamako, Kayes, Ségou and Mopti) except Sikasso; it represented 31.57 % of all the patterns (Table 4). Also, a unique pattern was observed in Mopti, which is the SBXXX mopti. The characteristic of this pattern is that, the spacers 4, 5, 22 and 23 are lacking (Table 3). In addition, the lack of the spacer 32 led to the spoligotype pattern SBXXXX bamako and found only in Bamako. In addition, we found that spoligotype patterns SB2262 and SB2263 lacked both spacers 6 and 21 (Figs. 2 and 3). These strains were found only in Bamako and Mopti.

Among the 67 specimens obtained from individual human subjects infected with pulmonary tuberculosis, only one had confirmed *M. bovis.* This case was also a pulmonary tuberculosis infection*.* In comparison to the spoligotypes from cattle, the spoligotype pattern obtained from human host was similar to the pattern observed in the cattle *M. bovis* pattern SB0300. Both lack the spacers 6 and 30 *(*Table 4)*.* The patient with the SB0300 spoligotype pattern of *M. bovis* had developed active tuberculosis with characteristic symptoms including weight loss, cough and fever. The serological testing for HIV was negative.

## Discussion

The estimated frequency of lesion was 11.70 % (79/675) with a disparity among regions. The highest lesion rate was observed in Kayes in the Western part of the country with 14.7 % whereas it was two times less in Sikasso in the Southern region with 7.5 % (Table 2). In contrast, the infection rate of *M. bovis* presents another profile; the overall frequency was 2.37 % (16/675) with the microscopy and 2.81 % (19/675) with the use of liquid media culture (Table 5). The highest frequency of *M. bovis* infection (by culture) was observed in Bamako and Mopti with respectively 3.63 % (8/220) and 3.57 % (5/140). The lowest frequency was found in Ségou with 1.25 % and in Kayes (1.33 %) where the lesion rate was higher (Table 1). There is then an indication that all the lesions were not due to *M. bovis* infection. Mopti and Bamako are two important places for livestock, Mopti is an excellent place in terms of cattle breeding, and Bamako is the big market for big consumption. Thus, the risk seems higher based on those data, it is likely that out of 100 cattle there is risk that at least two could be infected with *M. bovis.*

**Table 5:** Results of the smear microscopy from tissue pellets

Microscopy (AFB-Smear)	Number (*N*)	Percentage (%)
Positive Negative	16 63	20.3 79.7
Total	79	100

By indirect Auramine-Rhodamine (smear from tissue pellets), 20.3 % of the samples were positive by microscopy

Sixty-three percent of specimens from tuberculosis lesions were culture-positive. This proportion is similar to the frequency observed in Chad, which was 65 % as reported by Diguimbaye-Djaibé & al. in 2006 [11]*.* The smear positivity was only one fifth (20.3 %) of the specimens, which was low and confirms the lack of sensitivity of smear microscopy for tuberculosis diagnosis in this setting.

The frequency of BTB varies from one geographical zone to another depending on the population density of cattle. An example is Tanzania, where the prevalence of BTB (by using a single tuberculin skin test) was 14 % in the Southern Highlands [12], and 0.2 % in the Lake Victoria zone [13]*.* This difference was explained partially by the density and number of cattle in the region.

Tuberculosis in cattle has been demonstrated to be prevalent in sedentary breeding style, where cattle are used for milk production as these conditions allow better transmission of *M. bovis* infection between animals [14, 15]*.* In Mali, the prevalence of tuberculin-skin test positive cattle in Sikasso and Ségou was respectively 20 % and 64.3 % in different herds,1 % and 2.8 % in individual cattle, and the same observation was seen in the suburban area of Bamako in 2003 [14, 16]*.*

We further found that 10.1 % of specimens were infected with non-tuberculous mycobacteria (NTM) and 29.1 % with bacteria or yeast. Almost one third (36.7 %) of the specimens was culture negative, a proportion higher than what was seen in Chad (27.3 %) [11]. The isolation of NTM from tissue samples may represent true infections, co-infections or colonization. However, we do not know from our data the importance and infectiousness of those isolates. In view of the lack of pasteurization of milk, these data do suggest that, cattle may be one source of NTM transmission*.*

Our study was limited to the south of the country, and the cattle were from the urban areas of each region. The transmission profiles in rural areas of the regions may be different if we were able to include cattle from these areas. Thus, a cross sectional with larger sample size and sampling combining slaughterhouses, rural and urban areas from all the country may reveal the true country profile of *M.bovis* strains circulating in Mali. Another limitation was that our focus was mainly for identification of *M.bovis* and we didn’t focus on identifying other mycobacteria species. Despite these limitations, the study revealed that the Bamako region has the highest frequency of BTB and showed a wide variety of *M. bovis* strains (SB2262, SB2263, SB2264, and SB0300). Only the SB0300 was previously reported [8]. This frequency is higher than the one reported by Sidibé in 2003 [14].

Such high prevalence and diversity in Bamako, the capital city of Mali, may be due to the semi-intensive breeding style used in this region to improve milk and meat production. The high demand for meat in the capital city of Bamako increases the different varieties of cattle, the biggest market for livestock in Mali [14]*.* These data are in line with previous studies that showed that imported cattle are sensitive to different types of diseases in general and bovine tuberculosis in particular [14]*.* Another explanation may be the high number of tissue samples collected from Bamako region in comparison to the other regions (27/79), although this difference was not significant (*p* = 0.47). Bamako was followed by the Mopti region, which has the highest number of cattle in Mali and is an excellent geographical breeding zone because of its diverse climate [14]*.* We discovered a wide variation in *M. bovis* strains with five new spoligotype patterns identified. However, the particular distribution of the spoligotype SB0944 in Sikasso has to be investigated further. In fact, this region in the south of the country has received funding to breed the *N’dama race,* which is Trypano- tolerant [17]*.* In addition, all the strains observed in Sikasso had the spacer 6 which was absent in all strains studied so far in Mali [8]. This may explain the clustering of this spoligotype in Sikasso and we believe that strain was imported through the intense breeding related to this N’dama project*.* We also have identified seven cattle infected with *M. bovis* that are lacking the spacers 6 and 21. This is the first time those spoligotypes have been observed in Mali. In the previous study done in the Bamako abattoir, Muller & al. [8], have identified spoligotypes lacking spacer 6 in Bamako. We observed that five cattle were infected with strains of *M. bovis* lacking simultaneously the spacers 6 and 21, which may indicate that the genome is flexible enough to allow creation of new spoligotypes that in turn helps to increase the antigenic repertoire within *M. bovis*. The cluster number six was the most prevalent and was seen in four regions out of five [8].

All our examined specimens showed an absence of spacer 30 (Fig. 2), which confirmed the results obtained in 2007 by Muller et al. [8]. However, Muller found the presence of the spacer 30 among seven strains out of 20 from Bamako abattoir [18]. Taken together, *M. bovis* strains from Cameroun, Nigeria, Chad and Mali examined by spoligotyping technique showed similar spoligotype patterns that lacked the spacer 30 [18]. This may suggest that strains from those countries are phylogenetically similar [18]. The reason may be a specific deletion within a chromosomal DNA (Af 1) [18]. The shortest sequence through the Direct Repeat region was observed in the SBXXXX Mopti. In addition to known missing spacers, there are the spacers 4, 5, 22 and 23 which were also missing. The deletion of those spacers which are contiguous may have a rich AT content which is prone for deletion. During this study, *M. caprae*, which is known to be associated tuberculosis infection in sheep and goats, was not observed [19].

The use of spoligotyping for accurate diagnosis is important for assessing the fight against this zoonosis [20]. In this study, spoligotyping was used as a diagnostic and confirmatory tool, but also yielded important insight into the epidemiology and characteristic of *M. bovis* in Mali and revealed that more spoligotyping patterns are emerging. Another important impact of this study concerns the neighbor countries of Mali, since a large percentage of meat from cattle in Mali is consumed in those countries, and the mobility of livestock in the vast region of Sahel which could potentially lead to further transmission of BTB [4].

In this study, we identified one case of *M. bovis* infection in humans for a crude estimated prevalence of 1.5 % (1 out 67). This zoonotic TB was found in several countries in Africa with a median of 2.8 % (range 0–37.7 %). Our prevalence falls in that range and below the prevalence observed in Ethiopia, Nigeria and Tanzania [12, 21, 22]. We assume that the proportion of human cases of *M. bovis* depends on the efficacy of disease control such as regular milk pasteurization, and slaughterhouse meat inspection [10, 23]. In Mali, little is known about the transmission of *M. bovis* to, or between humans. *Mycobacterium bovis* can be easily transmitted through ingestion of unpasteurized milk, or undercooked meat and can cause pulmonary tuberculosis mainly in children [5, 12] among workers from farms and slaughterhouses [24]. Although we have not performed a proper study on risk factors analysis, but we may speculate that those means are the common route of *M. bovis* among our patient.

## Conclusion

*M. bovis* is present in Mali and accounts for approximately one quarter of suspected lesions from infected cattle in the tested regions. Our approach by the use of spoligotyping is a logical step for a better understanding of different mycobacterial species circulating in the country. In order to further characterize the frequency of BTB in Mali and to estimate human transmission rates of NTM as well as BTB, more extensive testing is necessary. The presence of confirmed *M. bovis* in one Malian subject suggests that there may be a lack of effective disease control which includes regular milk pasteurization and slaughterhouse meat inspection.

## Abbreviations

BTB, bovine tuberculosis; DR, direct repeat; ECL, enhanced chemiluminescent; GDP, Growth Domestic Product; HIV, human immunodeficiency virus; IS6110 RFLP, restriction fragment length polymorphism; MIRU, Mycobacterial Interspersed Repetitive Units; MTBc, Mycobacterium tuberculosis complex; NTM, non-tuberculous mycobacteria; TB, tuberculosis; USD, Dollar of United States of America; USTTB, University of Sciences, Techniques and Technologies of Bamako; VNTR, variable number of tandem repeats
